# Inhibition of hydrogen uptake in *Escherichia coli *by expressing the hydrogenase from the cyanobacterium *Synechocystis *sp. PCC 6803

**DOI:** 10.1186/1472-6750-7-25

**Published:** 2007-05-23

**Authors:** Toshinari Maeda, Gönül Vardar, William T Self, Thomas K Wood

**Affiliations:** 1 Artie McFerrin Department of Chemical Engineering 220 Jack E. Brown Building, Texas A & M University College Station, TX 77843-3122, USA; 2 Department of Biology, Texas A & M University College Station, TX 77843-3258, USA; 3 Zachry Department of Civil and Environmental Engineering, Texas A & M University College Station, TX 77843-3136, USA; 4 Department of Molecular Biology and Microbiology, Room 124 Biomolecular Science Center Bldg. 20,4000 Central Florida Blvd., University of Central Florida, Orlando, FL 32816, USA

## Abstract

**Background:**

Molecular hydrogen is an environmentally-clean fuel and the reversible (bi-directional) hydrogenase of the cyanobacterium *Synechocystis *sp. PCC 6803 as well as the native *Escherichia coli *hydrogenase 3 hold great promise for hydrogen generation. These enzymes perform the simple reaction 2H^+ ^+ 2e^- ^↔ H_2 _(g).

**Results:**

Hydrogen yields were enhanced up to 41-fold by cloning the bidirectional hydrogenase (encoded by *hoxEFUYH*) from the cyanobacterium into *E. coli*. Using an optimized medium, *E. coli *cells expressing *hoxEFUYH *also produced twice as much hydrogen as the well-studied *Enterobacter aerogenes *HU-101, and hydrogen gas bubbles are clearly visible from the cultures. Overexpression of HoxU alone (small diaphorase subunit) accounts for 43% of the additional hydrogen produced by HoxEFUYH. In addition, hydrogen production in *E. coli *mutants with defects in the native formate hydrogenlyase system show that the cyanobacterial hydrogenase depends on both the native *E. coli *hydrogenase 3 as well as on its maturation proteins. Hydrogen absorption by cells expressing *hoxEFUYH *was up to 10 times lower than cells which lack the cloned cyanobacterial hydrogenase; hence, the enhanced hydrogen production in the presence of *hoxEFUYH *is due to inhibition of hydrogen uptake activity in *E. coli*. Hydrogen uptake by cells expressing *hoxEFUYH *was suppressed in three wild-type strains and in two *hycE *mutants but not in a double mutant defective in hydrogenase 1 and hydrogenase 2; hence, the active cyanobacterial locus suppresses hydrogen uptake by hydrogenase 1 and hydrogenase 2 but not by hydrogenase 3. Differential gene expression indicated that overexpression of HoxEFUYH does not alter expression of the native *E. coli *hydrogenase system; instead, biofilm-related genes are differentially regulated by expression of the cyanobacterial enzymes which resulted in 2-fold elevated biofilm formation. This appears to be the first enhanced hydrogen production by cloning a cyanobacterial enzyme into a heterologous host.

**Conclusion:**

Enhanced hydrogen production in *E. coli *cells expressing the cyanobacterial HoxEFUYH is by inhibiting hydrogen uptake of both hydrogenase 1 and hydrogenase 2.

## Background

Cyanobacteria are diverse, ancient (present 3.5 billion years ago), photosynthetic, and photoautotrophic, and it is believed that these bacteria evolved to become chloroplasts in plant cells [1]. Cyanobacteria have at least three enzymes involved in hydrogen synthesis/metabolism: (i) nitrogenase which produces hydrogen as nitrogen is converted to ammonia, (ii) uptake hydrogenase which consumes hydrogen produced by nitrogenase, and (iii) a bi-directional hydrogenase which can both consume and produce hydrogen [1]. The bi-directional hydrogenase was employed here since hydrogen production via the nitrogenase requires substantially more energy from the cell (16 ATP per mole of hydrogen) so it would be a less-energy-efficient system (N_2 _+ 8H^+ ^+ 8e^- ^+16ATP → 2NH_3 _+ H_2 _+ 16ADP + 16P_i_) [1].

The reversible (bi-directional) hydrogenase enzyme of *Synechocystis *sp. PCC 6803 produces hydrogen via the reaction 2H^+ ^+ 2e ↔ H_2 _(g) [1]; the source of the two electrons is NADH. The genes (*hoxEFUYH*) encoding this enzyme were identified and indicate that HoxFU are iron-sulfur proteins that bind NADH (diaphorase) and that the large hydrogenase subunit HoxH contains six conserved sites for binding the Ni-Fe cofactor [2]. The small hydrogenase subunit HoxY may bind a [4Fe-4S] cluster [1]. The function of HoxE is not clear but it may be a bridging subunit in the membrane [3]. We chose this bacterium since it is well-characterized with the complete genome (3,573,470 bp) sequenced in 1996 [4]; hence, the hydrogenase is readily cloned. Transcription of HoxEFUYH is regulated by the LexA transcription activator, which specifically binds to the promoter region of the *hox *operon [5,6]. Note that the hydrogenase enzyme is sensitive to oxygen [7], so the assays are performed anaerobically.

Hydrogenase enzymes in *E. coli *are involved in two distinct modes of hydrogen metabolism: hydrogen production via hydrogenase 3 and hydrogen uptake by hydrogenases 1 and 2 [8]. Hydrogenase 1 (encoded by *hyaABCDEF*), hydrogenase 2 (encoded by *hybOABCDEFG*), and hydrogenase 3 (encoded by *hycABCDEFGHI*) have nickel, iron, and three non-protein diatomic ligands (cyanide and carbon monoxide) in the active site which rely on the auxiliary proteins HypABCDEF (metalochaperones for NiFe insertion) and SlyD (nickel insertion) for maturation as well as may possibly rely on the chaperones GroEL/GroES [9]. Hydrogenase 1 and hydrogenase 2 are *αβ *heterodimers of a small subunit and a Ni-Fe containing catalytic large subunit and are present in the inner membrane facing the periplasmic space [10-12]. In *E. coli*, hydrogen is produced by hydrogenase 3 in the formate hydrogenlyase system (FHL) [13]. *hycE *encodes the large subunit of hydrogenase 3, and *hycA *encodes the repressor gene of the FHL system including the *hyc *operon [14]. HycI protease catalyses a C-terminal proteolytic cleavage of the HycE large subunit, and HypA, HypB, HypC, HypD, HypE, and HypF are required for metallocenter assembly [15]. Ordinarily, cyanobacteria employ photosynthesis fueled by light energy to produce hydrogen. However, if an active hydrogenase from a cyanobacterium may be expressed in *E. coli*, it is possible to use the energy from simple sugars (e.g., from agricultural products and wastes) to produce hydrogen. Other advantages of using *E. coli *are that the use of energy from sugar rather than light avoids relying on the availability of light and avoids the production of oxygen as occurs during photosynthesis. Oxygen as an impurity in hydrogen arising from photosynthetic activity is undesirable for fuel cells based on enzyme electrodes [16] and is undesirable as a fire hazard [17]. Hence, large production of hydrogen is more advantageous via fermentation rather than photochemical production [17].

Hydrogen is a 100% renewable fuel that burns cleanly, is efficient, and generates no toxic by-products [7]. Hydrogen is also the preferred choice for fuel cells. Not only is H_2 _a clean fuel, producing only water as its by-product, it actually has a higher energy content than oil (142 MJ/kg for H_2 _vs. 44.2 MJ/kg for oil), and is thus more efficient. Most of the H_2 _now produced globally is by the process of steam reforming and the water-gas shift reaction, or as a by-product of petroleum refining and chemicals production [18]. Use of biological methods of H_2 _production promises significant energy reduction costs, as these processes do not require extensive heating (or extensive electricity as in electrolysis plants). Here we report the cloning of an active cyanobacterial enzyme complex into *E. coli *to enhance hydrogen production primarily by limiting hydrogen uptake by the native *E. coli *hydrogenases.

## Results

### Enhanced *E. coli *hydrogen production by HoxEFUYH

**Figure 1:**
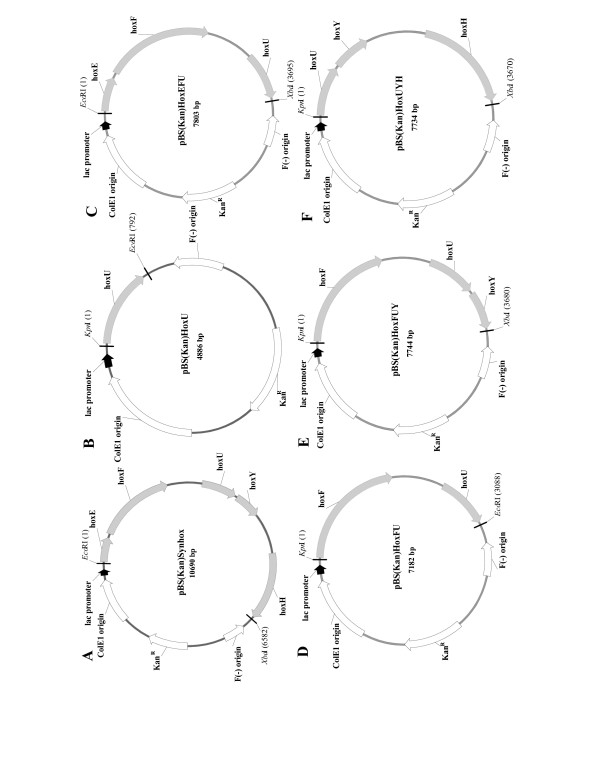
Vectors pBS(Kan)Synhox (A), pBS(Kan)HoxU (B), pBS(Kan)HoxEFU (C), pBS(Kan)HoxFU (D), pBS(Kan)HoxFUY (D), and pBS(Kan)HoxUYH (E). Kan^R ^is the kanamycin resistance gene. The five genes coding for the hydrogenaseare *hoxEFUYH*.

To create a recombinant system which produces hydrogen via fermentation, we cloned the hydrogenase locus (*hoxEFUYH*) of *Synechocystis *sp. PCC 6803 into the well-studied bacterium *E. coli*. DNA sequencing and restriction enzyme digests showed the correct locus was cloned; our plasmid was designated pBS(Kan)Synhox (Figure 1A).

**Figure 2:**
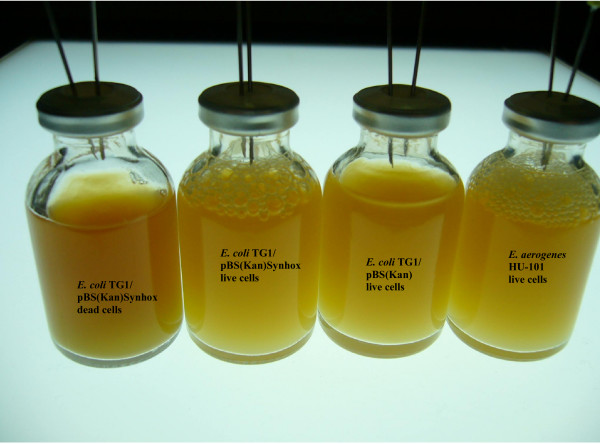
Hydrogen production with *E. coli *TG1/pBS(Kan)Synhox and *E. aerogenes *HU-101 (hydrogen bubbles shown). Representative samples shown from hydrogen assay experiments in complex medium with glucose (repeated 3 times).

Native *E. coli *TG1 produces hydrogen via the FHL system during mixed-acid fermentations [19], and TG1 expressing the cyanobacterial hydrogenase produced 3-fold more hydrogen than cells which lacked the *hoxEFUYH *locus (22 ± 1 vs. 7 ± 1 μmol/mg protein) after 6 h in complex medium. More hydrogen was measured at least 23 times for cells expressing *hoxEFUYH *relative to the negative control that lacks the cyanobacterial locus so the effect is reproducible. The negative controls of both autoclaved TG1/pBS(Kan)Synhox and autoclaved *E. aerogenes *HU-101 did not produce hydrogen. Note that co-elution with pure hydrogen confirmed that hydrogen was produced by the *E. coli *cells and also our retention time for hydrogen was consistent with literature values (22.8 vs. 22 sec) [20]. In addition, the recombinant *E. coli *expressing the cyanobacterial hydrogenase produced about 2-fold more hydrogen after 6 h in complex medium than the positive control *E. aerogenes *HU-101 (11 ± 0.3 μmol/mg protein), a well-studied producer of hydrogen [21,22]. Furthermore, hydrogen gas bubbles were clearly more visible in the recombinant strain compared to the host which lacked the cyanobacteria locus and also visible in the positive control but were not visible with autoclaved samples (Figure 2). Therefore, the recombinant *E. coli *strain produces significantly higher quantities of hydrogen gas.

### Optimization of medium and time course of hydrogen production

The cyanobacterial genes were fused to a *lac *promoter in pBS(Kan); therefore, the expression of *hoxEFUYH *will be suppressed by catabolite repression if glucose is included in complex medium. To search for an optimal medium for producing hydrogen, glucose was replaced with fructose, galactose, maltose, lactose, glycerin, citrate, and succinate in complex medium. Hydrogen production in complex medium with fructose, galactose or maltose was 20% more than that with glucose in TG1/pBS(Kan)Synhox whereas the other carbon sources did not improve hydrogen production.

**Figure 3:**
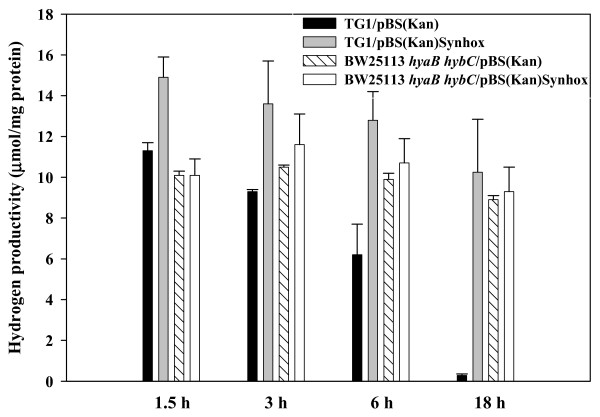
Time course of hydrogen productivity (via gas chromatography) incomplex medium for TG1/pBS(Kan), TG1/pBS(Kan)Synhox, BW25113 *hyaB hybC*/pBS(Kan), and BW25113 *hyaB hybC*/pBS(Kan)Synhox.

To investigate hydrogen production with TG1/pBS(Kan)Synhox in more detail, a hydrogen time course experiment was performed. Hydrogen produced by *E. coli *TG1 cells with or without HoxEFUYH was maximum within 1.5 h (Figure 3). Furthermore, from 1.5 h to 6 h, hydrogen produced in the absence of HoxEFUYH decreased 2.1 ± 0.4-fold more rapidly than that from cells with HoxEFUYH (Figure 3); hence, the hydrogen formed in the presence of HoxEFUYH was more stable. This suggested that hydrogen uptake is inhibited by expression of active HoxEFUYH. Note that after 18 h, the hydrogen yield from cells expressing *hoxEFUYH *was over 41 times more than that of the wild-type strain (Figure 3).

### Enhanced hydrogen production depends on native hydrogenase 3

To ascertain if elements of the native *E. coli *host FHL system impact the cyanobacteria hydrogenase system, hydrogenase activities of the cyanobacteria and the *E. coli *FHL were assayed in a series of mutants that lack either *E. coli *hydrogenase 3 of the FHL (HD705) or lack the maturation machinery required for assembling hydrogenases in *E. coli *(SE1497 and PMD23). We also examined a mutant that lacked the transcriptional activator FhlA, and thus would not express genes encoding the FHL complex (SE1174) as well as assayed a mutant that cannot form selenoproteins (WL400) and thus is unable to produce active formate dehydrogenase H (formate dehydrogenase H is sole electron donor for hydrogenase 3). Since the parent strain MC4100 with pBS(Kan)Synhox produced hydrogen but the FHL mutants (HD705, SE1174, SE1497 PMD23, and WL400) harboring pBS(Kan)Synhox did not produce hydrogen gas, active expression of the cyanobacteria hydrogenase system relies on both an active *E. coli *hydrogenase 3 as well as the maturation proteins of the host and cannot simply be due to HoxEFUYH acting as an electron donor to HYD3.

### Role of the cyanobacterial proteins HoxEFUYH

**Figure 4:**
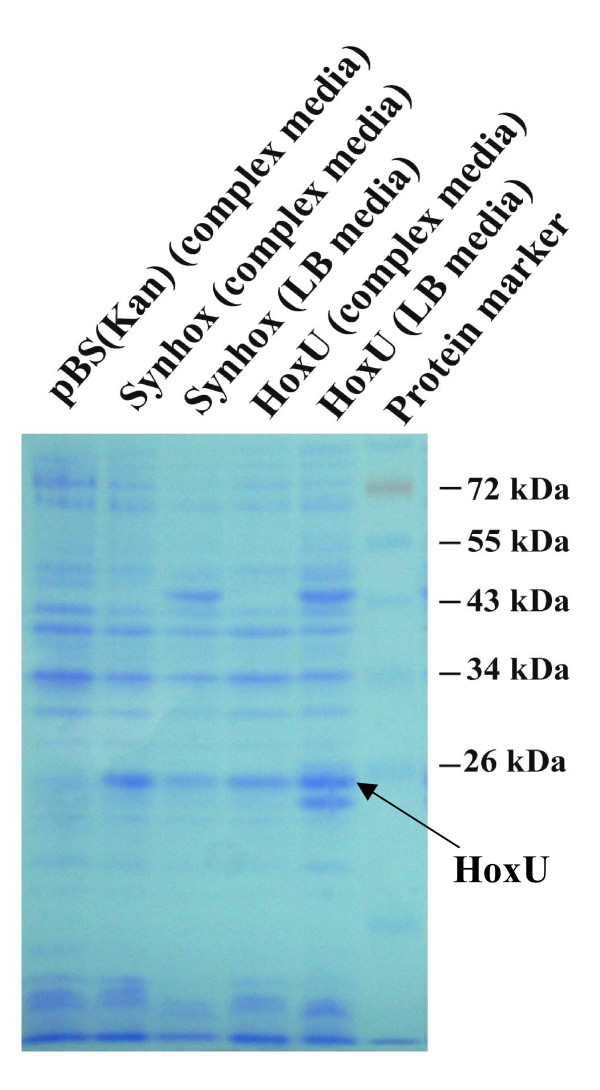
SDS-PAGE of HoxEFUYH and HoxU expression from *E. coli *TG1/pBS(Kan)Synhox and TG1/pBS(Kan)HoxU grown anaerobically in complex media and LB media. Arrow indicates the expression of HoxU. pBS(Kan) is TG1/pBS(Kan) (negative control), and Synhox is TG1/pBS(Kan)Synhox.

To observe the expression of the recombinant enzymes, SDS-PAGE was performed. As shown in Figure 4, HoxU (26 kDa) from the cyanobacterium was clearly expressed in TG1/pBS(Kan)Synhox that produced hydrogen gas in both complex medium and LB medium while the other proteins (HoxE, 18 kDa; HoxF, 62 kDa; HoxY, 23 kDa; HoxH, 51 kDa) were not observed. As expected, HoxU was not observed in TG1/pBS(Kan) (negative control). Also, the expression of HoxU was greater in complex medium relative to LB medium and complex medium produced more hydrogen. Hence, HoxU of *Synechocystis *sp. PCC 6803 may play a major role in the elevated hydrogen production in *E. coli*.

To discern if only HoxU is required for producing more hydrogen, *hoxU *was cloned under the *lac *promoter (designated as pBS(Kan)HoxU, Figure 1B), and hydrogen production in TG1 harboring pBS(Kan)HoxU was examined. TG1/pBS(Kan)HoxU produced more hydrogen than TG1/pBS(Kan) but only 44.6 ± 7.3% of that of TG1/pBS(Kan)Synhox. These results indicate that the additional hydrogen produced by expressing HoxEFUYH is not solely the result of active HoxU; hence, other active HoxEFUYH proteins are required for producing hydrogen.

To determine the importance of the other cyanobacterial enzymes for hydrogen production, pBS(Kan)HoxEFU (Figure 1C), pBS(Kan)HoxFU (Figure 1D), pBS(Kan)HoxFUY (Figure 1E), and pBS(Kan)HoxUYH (Figure 1F) were also constructed and hydrogen production in TG1 with these plasmids was tested. Hydrogen production in TG1/pBS(Kan)HoxEFU and TG1/pBS(Kan)HoxFU showed 48 ± 7% and 57 ± 2% of the hydrogen produced by TG1/pBS(Kan)Synhox; hence, these strains had about half the hydrogen produced by cloning either HoxEFUYH or the almost same value as that by expressing only HoxU and they confirm the importance of HoxU. In contrast, TG1/pBS(Kan)HoxFUY and TG1/pBS(Kan)HoxUYH produced 81 ± 22% and 113 ± 31% of the hydrogen produced by TG1/pBS(Kan)Synhox, respectively; therefore, hydrogen production in TG1/pBS(Kan)HoxUYH was somewhat better than that in TG1/pBS(Kan)Synhox indicating the importance of proteins HoxU, HoxY, and HoxH.

### Mechanism of enhanced hydrogen production is via inhibition of hydrogen uptake

Since the hydrogen time course experiment (Figure 3) showed the hydrogen produced by cells expressing *hoxEFUYH *was more stable than hydrogen from cells which lacked this locus and given that the greater hydrogen production seen upon cloning the cyanobacterial locus was dependent on both the native hydrogenase maturation proteins and active hydrogenase 3, we theorized that HoxEFUYH may be influencing hydrogen uptake. Recall that hydrogenase 1 and 2 are involved in hydrogen uptake only [10,11]. Hence, hydrogen uptake activity was measured three ways to investigate this hypothesis. As shown in Table 1, *E. coli *TG1 expressing HoxEFUYH had 3.3 times less hydrogen uptake compared to the negative control TG1/pBS(Kan) and *E. coli *MC4100 had similar results. These hydrogen uptake results were corroborated by using a plate assay for reversible hydrogenase activity which showed 10-fold less hydrogen uptake upon expressing HoxEFUYH and by a GC-based hydrogen up-take assay which indicated that H_2 _uptake activity in TG1/pBS(Kan)Synhox is 2.1 ± 0.2-fold less than TG1/pBS(Kan) over 0 to 6 h. Hence, the active cyanobacterial enzymes (HoxEFUYH) inhibit hydrogen uptake consistently in *E. coli*.

**Table 1:** Hydrogen uptake activity with various *E. coli *BW25113 mutants in complex medium as determined by the oxidized methylviologen-based H_2 _uptake assay after 5 min

**Strain**	**Description**	**Hydrogen uptake**	
		
		**nmol/min/mg protein**^a^	**relative**
*E. coli *BW25113/pBS(Kan)	wild type	94 ± 12	3.3
*E. coli *BW25113/pBS(Kan)Synhox	wild type + HoxEFUYH	29 ± 7	1
*E. coli *BW25113 *hyaA*/pBS(Kan)Synhox	*ΔhyaA *(defective hydrogenase 1) + HoxEFUYH	59 ± 21	2.0
*E. coli *BW25113 *hyaB*/pBS(Kan)Synhox	*ΔhyaB *(defective hydrogenase 1) + HoxEFUYH	59 ± 6	2.0
*E. coli *BW25113 *hybB*/pBS(Kan)Synhox	*ΔhybB *(defective hydrogenase 2) + HoxEFUYH	73 ± 13	2.5
*E. coli *BW25113 *hybC*/pBS(Kan)Synhox	*ΔhybC *(defective hydrogenase 2) + HoxEFUYH	80 ± 11	2.8
*E. coli *BW25113 *hycE*/pBS(Kan)	*ΔhycE *(defective hydrogenase 3)	25 ± 0.8	0.9
*E. coli *BW25113 *hycE*/pBS(Kan)Synhox	*ΔhycE *(defective hydrogenase 3) + HoxEFUYH	7 ± 2	0.3
*E. coli *BW25113 *hycG*/pBS(Kan)Synhox	*ΔhycG *(defective hydrogenase 3) + HoxEFUYH	10 ± 2	0.4
*E. coli *BW25113 *hyaB hybC*/pBS(Kan)	*ΔhyaB* and *ΔhybC *(defective hydrogenase 1 and 2)	21 ± 1	0.7
*E. coli *BW25113 *hyaB hybC*/pBS(Kan)Synhox	*ΔhyaB* and *ΔhybC *(defective hydrogenase 1 and 2) + HoxEFUYH	21.6 ± 0.3	0.8
*E. coli *BW25113 *hyaB hybC hycE*/pBS(Kan)	*ΔhyaB*, *ΔhybC*, and *ΔhycE *(defective hydrogenase 1, 2, and 3)	1.8 ± 0.4	0.06
*E. coli *MC4100/pBS(Kan)	wild type	35 ± 15	2.3
*E. coli *MC4100/pBS(Kan)Synhox	wild type + HoxEFUYH	15 ± 6	1
*E. coli *TG1/pBS(Kan)	wild type	16 ± 7	3.3
*E. coli *TG1/pBS(Kan)Synhox	wild type + HoxEFUYH	5 ± 3	1
*E. coli *HD705/pBS(Kan)	defective hydrogenase 3	8 ± 1	5.4
*E. coli *HD705/pBS(Kan)Synhox	defective hydrogenase 3 + HoxEFUYH	2 ± 2	1

Standard deviations shown from one representative experiment with 2 replicates. a IPTG was added for hydrogen uptake (1 mM) assays activity for 6 h in complex medium.

To determine by which of the three native *E. coli *hydrogenases that hydrogen uptake was affected by HoxEFUYH, a series of isogenic mutants of *E. coli *BW25113 was used (Table 1). Using two mutants for hydrogenase 1 (*hyaA* and *hyaB*), hydrogen uptake was measured and found to increase consistently 2-fold upon removing hydrogenase 1 in the presence of HoxEFUYH; hence, HoxEFUYH decreases hydrogen uptake via hydrogenase 1. Similarly, using two mutants for hydrogenase 2 (*hybB* and *hybC*), hydrogen uptake increased consistently 2.5- to 2.8-fold (Table 1) upon removing hydrogenase 2 in the presence of HoxEFUYH; hence, HoxEFUYH decreases hydrogen uptake via hydrogenase 2. In contrast, upon adding HoxEFUYH, isogenic mutations that eliminate hydrogenase 3 activity (*hycE *and *hycG*) (Table 1) decreased hydrogen uptake (rather than increasing it). In addition, hydrogen uptake in the *hyaB hybC* double mutant that eliminated hydrogenase 1 and 2 activity was identical to that of the wild-type strains expressing *hoxEFUYH *and the expression of *hoxEFUYH *had no effect in the double mutant (Table 1). Corroborating these results, hydrogen production by BW25113 *hyaB hybC*/pBS(Kan)Synhox was the same as BW25113 *hyaB hybC*/pBS(Kan) (Figure 3). On the other hand, a triple mutant (*hyaB hybC hycE*; defective in hydrogenase 1, 2, and 3) with pBS(Kan) or pBS(Kan)Synhox did not produce hydrogen (data not shown), indicating that hydrogenase 3 is essential for producing hydrogen. Therefore, HoxEFUYH works through hydrogenase 1 and hydrogenase 2 rather than through hydrogenase 3, and the decrease in hydrogen uptake seen with the hydrogenase 3 mutants is due to HoxEFUYH inhibition of the remaining active hydrogenase 1 and 2 enzymes.

### DNA microarrays

To investigate whether cloning of the cyanobacterial *hoxEFUYH *merely increased hydrogen production by up-regulating the native *E. coli *hydrogenase system, we examined differential gene expression upon expression of *hoxEFUYH *from pBS(Kan)Synhox. The microarrays showed that gene expression for the *hya, hyb, hyc*, and *hyp *operons was not altered between TG1/pBS(Kan)Synhox and TG1/pBS(Kan); hence, functional HoxEFUYH is necessary for enhanced hydrogen production in *E. coli *(Additional file 1). Surprisingly, the differential gene expression indicated that primarily biofilm-related genes are regulated by expressing HoxEFUYH as shown in Table 2. To investigate the effect of expression of HoxEFUYH on biofilm formation, a 96-well polystyrene plate assay was performed. *E. coli *TG1 expressing HoxEFUYH produced 1.7 ± 0.2 times more biofilm than TG1/pBS(Kan) under anaerobic conditions in LB with 0.2% glucose after 48 h at 37°C.

**Table 2:** Differentially-expressed, biofilm-related genes upon expression of HoxEFUYH in *E. coli *TG1 in complex medium at 37°C after 6 h. References indicate the relevant biofilm publication

**Gene name**	**b #**	**Fold change**	**Gene function**	**Reference**
*ydfX*	b1568	34.3	hypothetical protein	[31]
*tnaL*	b3707	24.3	tryptophanase leader peptide	[31, 34]
*tnaA*	b3708	13.9	tryptophanase	[31]
*sdhC*	b0721	10.6	succinate dehydrogenase membrane protein	[31, 32]
*sdhD*	b0722	9.2	succinate dehydrogenase membrane protein	[32, 34]
*treB*	b4240	9.2	subunit of EIITre	[34]
*treC*	b4239	6.1	trehalose-6-phosphate hydrolase	[31-33]
*pspA*	b1304	9.2	regulatory protein for the phage shock protein operon	[32]
*pspB*	b1305	8.0	stimulates PspC-mediated transcriptional activation of the *psp *operon	[32]
*yhaN*	b3109	8.6	hypothetical ORF	[31]
*tdcC*	b3116	8.6	threonine STP transporter	[32]
*tdcD*	b3115	8.0	propionate kinase/acetate kinase C	[32]
*gltA*	b0720	8.0	subunit of citrate synthase	[32]
*yciD*	b1256	8.0	subunit of Colicin S4 Transport System	[31]
*gatA*	b2094	6.1	subunit of EIIGat	[31, 34]
*gatB*	b2093	7.5	subunit of EIIGat	[31]
*ibpB*	b3686	6.5	small heat shock protein	[33, 34]
*nmpC*	b0553	6.1	outer membrane porin protein	[31, 34]
*Mdh*	b3236	5.7	subunit of malate dehydrogenase	[32]
*sucC*	b0728	5.3	succinyl-CoA synthetase, βsubunit	[32]
*fumB*	b4122	- 14.9	subunit of fumarase B	[31-33]
*yigE*	b3814	- 11.3	conserved protein	[31, 32]
*ydeO*	b1499	- 10.6	transcriptional activator	[31, 32]
*yjjJ*	b4385	- 9.2	conserved protein	[31, 32]
*yihP*	b3877	- 8.0	GPH transporter	[32, 33]
*yjeN*	b4157	- 8.0	conserved protein	[32]
*yfjT*	b2637	- 7.5	CP4-57 prophage predicted protein	[32]
*yjhS*	b4309	- 6.5	conserved protein	[32]
*yghG*	b2971	- 5.3	unknown function	[32, 33]
*yicE*	b3654	- 5.3	NCS2 transporter	[31, 32]
*tort*	b0994	- 3.0	unknown inducer	[32-34]

## Discussion

To date, no cyanobacterial hydrogenase protein has been actively expressed in *E. coli*. Functional expression here of the cyanobacterial hydrogenase components in *E. coli *allows for both mutagenesis for structure/function determinations as well as for enhanced hydrogen production via saturation mutagenesis and DNA shuffling [23]. Also, *E. coli *cells offer two advantages over normally photosynthetic microbes regarding protein evolution. First, the transformation efficiency of *E. coli *(greater than 10^9 ^transformants per microgram of plasmid) is at least three orders of magnitude greater than those of photosynthetic bacteria [24]. A rapid *E. coli*-based genetic selection method for hydrogenase activity would enable the sampling of thousands of different hydrogenase mutants in a single day. Second, hydrogen production by cyanobacteria requires light, and oxygen is produced through photosynthesis; oxygen production is undesirable for fuel cells; therefore, it is more desirable to clone the hydrogenase into *E. coli *rather than cyanobacteria since *E. coli *is a facultative anaerobe.

In cyanobacterium *Synechocystis *sp. PCC 6803, HoxFU are iron-sulfur proteins that bind NADH (diaphorase) [25]. Also, HoxU is thought to serve as the bridging unit in the link between respiration and the hydrogenase [26]. In addition, cyanobacteria *Anacystis nidulans *SAUG 1402-1 and *Anacystis *sp. PCC 7942 showed reduced hydrogen-evolution catalyzed by the bidirectional hydrogenase upon mutation of *hoxU *[27]; these indicate that HoxU is important for hydrogenase activity. Our SDS-PAGE results demonstrated that the HoxU protein (26 kDa) was clearly expressed in TG1/pBS(Kan)Synhox and that the expression of HoxU increased according to increasing hydrogen production. Since only HoxU was seen with SDS-PAGE, we surmised that it may have its own promoter. Corroborating this, between the stop codon of *hoxF *and the start codon of *hoxU *there is a gap of 705 bp that contains six putative promoters upstream of *hoxU *based on promoter prediction software [28]. The expression of HoxU is probably regulated independently by at least one of these promoters; this suggested that controlling expression of HoxU may provide significant insight for elevating hydrogen production, which proved correct since cloning only *hoxU *accounted for about half of the hydrogen produced by cloning *hoxEFUYH*. This shows the other cyanobacterial proteins (HoxEFYH), although not clearly observed with SDS-PAGE, are beneficial for producing hydrogen. Since expressing HoxUYH in *E. coli *TG1 yields nearly the same effect as expressing all of HoxEFUYH via TG1/pBS(Kan)Synhox, HoxUYH are clearly important for enhanced hydrogen production.

Hydrogen production was enhanced as much as 41-fold via production of the active cyanobacterial HoxEFUYH, and the hydrogen produced using TG1/pBS(Kan)Synhox was more stable in comparison to that of TG1/pBS(Kan); this indicated that TG1/pBS(Kan)Synhox has reduced hydrogen uptake activity. Since any mutation related to hydrogenase 3 eliminated the benefit of expressing the cyanobacterial hydrogenase, it is clear that hydrogenase 3 is necessary for the HoxEFUYH effect since only hydrogenase 3 produces hydrogen in *E. coli *whereas HoxEFUYH maintains this hydrogen that is produced by limiting hydrogen uptake by hydrogenase 1 and 2. In accordance with this interpretation, hydrogen uptake was found to be 5 to 10 times lower upon expression of HoxEFUYH. Also, the microarray analysis shows that native hydrogenase gene expression is not affected by enhanced hydrogen production with TG1/pBS(Kan)Synhox, so there are no transcriptional effects related to cloning *hoxEFUYH*. Taken together, these results show expression of HoxEFUYH increases hydrogen production by reducing hydrogen uptake of the native *E. coli *hydrogenases, but hydrogenase 3 is required to produce the hydrogen in the first place. Note that hydrogenase 1 and 2 have hydrogen uptake activity [10,11], and hydrogenase 3 has hydrogen production activity (hydrogenase 3 is the primary source of hydrogen gas production in *E. coli *[29] as hydrogenase 4 is inactive [30])

In contrast to the native hydrogenase gene expression, the DNA microarrays indicated the expression of many biofilm-related genes were altered upon expression of HoxEFUYH (Table 2), and this altered expression led to an increase in biofilm formation. Interestingly, our study of temporal gene expression in *E. coli *K-12 biofilms [31] shows that the genes related to hydrogenase 1 (*hyaABCDE*) and to hydrogenase 2 (*hybBC*) are transiently repressed, that the genes related to hydrogenase 3 (*hycBF*) and hydrogenase 4 (*hyfBC*) are up-regulated in the process of biofilm formation, and that some *hya *or *hyf *mutants produce 3- to 7-fold more biofilm. So the fact that expression of HoxEFUYH affects biofilm genes (Table 2) and that hydrogenase genes are routinely found in biofilm studies [31-34] suggest that biofilm formation is related to enhanced hydrogen production. Hence, it appears that biofilm formation may repress hydrogen uptake activity or induce hydrogen production, either of which would result in enhanced hydrogen production. In the future, we will need to ascertain whether biofilm formation is directly related to enhanced hydrogen production and how HoxEFUYH represses hydrogen uptake activity in *E. coli*.

To the best of our knowledge, on a protein basis, *Citrobacter *sp. Y19 (65 μmol/mg/h) [35], *Rhodopseudomonas palustris *JA1 (56 μmol/mg/h) [36], *Rhodopseudomonas palustris *P4 (41 μmol/mg/h) [37], and *Klebsiella oxytoca *HP1 (30 μmol/mg/h) [38] all have higher maximum hydrogen activity compared to our TG1/pBS(Kan)Synhox recombinant (6 μmol/mg/h); however, these organisms are more fastidious than *E. coli*. Therefore, *E. coli *holds promise for producing hydrogen as a more robust model host for heterologous expression of hydrogenases. Along these lines, Yoshida et al. [39] recently increased native hydrogenase expression in *E. coli *by inactivating the HycA FHL repressor and by overexpressing the FhlA transcriptional activator for the *hyc *and *hyp *operons with the result that 2.8-fold more hydrogen was generated (further increases, up to 250 μmol/mg/h, were obtained using a novel high-density reactor and formate addition). This approach suggests similar genetic changes (along with the aforementioned DNA shuffling) may be used to increase further hydrogen production using both the native and cyanobacterial hydrogenases.

## Conclusion

*E. coli *TG1 cells with pBS(Kan)Synhox yielded 41 times more hydrogen after 18 h than those with empty vector pBS(Kan) due to active HoxEFUYH from *Synechocystis *sp. PCC 6803 (primarily through active HoxUYH). The mechanism for this enhanced hydrogen production is that hydrogen is formed first by hydrogenase 3 (so the HoxEFUYH effect relies on active hydrogenase 3 and its maturation proteins), then HoxEFUYH inhibits hydrogen uptake by *E. coli *native hydrogenase 1 and hydrogenase 2. In effect, a novel way to reduce reversible hydrogen formation has been discovered using a cyanobacterial locus.

## Methods

### Bacterial strains, growth, and total protein

Strains are shown in Table 3. *E. coli *cells containing pBS(Kan) and derivatives were initially streaked from -80°C glycerol stocks on Luria-Bertani (LB) agar plates [40] containing 100 μg/mL kanamycin and incubated at 37°C. After growth on LB agar plates, these strains were cultured from a fresh single colony in LB medium [40] or complex medium [22] supplemented with 100 μg/mL kanamycin at 37°C with shaking at 250 rpm (New Brunswick Scientific Co., Edison, NJ). Wild-type *E. coli *K-12 BW25113 was obtained from the Yale University CGSC Stock Center, and its isogenic deletion mutants (Keio collection) were obtained from the Genome Analysis Project in Japan [41]. *Enterobacter aerogenes *HU-101 [22] was obtained from National Institute of Technology and Evaluation Biological Resource Center, Japan (accession number 100048) and was used as a positive control for hydrogen production; this strain was grown in complex medium. To optimize the medium for producing hydrogen, fructose (20 g, J.T. Baker Chemical, Phillipsburg, NJ), galactose (20 g, Aldrich Chemical, Milwaukee, WI), maltose (10 g, Sigma Chemical, St. Louis, MO), lactose (10 g, Sigma Chemical), glycerol (40 g, Fisher Scientific, Fair Lawn, NJ), citrate (30 g, Fisher Scientific) or succinate (40 g, Fisher Scientific) were substituted for glucose (20 g, Fisher Scientific) in complex medium. Plasmids pBS(Kan) [42] and pBS(Kan)Synhox (below) were electroporated into the formate hydrogenlyase mutants (Table 3). Cell growth was measured using turbidity at 600 nm, and total protein concentrations for *E. coli *and *E. aerogenes *HU-101 were 0.22 mg/OD/mL or 0.34 mg/OD/mL, respectively (Protein assay kit, Sigma Diagnostics, St. Louis, MO).

**Table 3:** Strains and plasmids used. Km^R^, Cm^R ^and Ap^R ^are kanamycin, chloramphenicol and ampicillin resistance, respectively.

**Strains and plasmids**	**Genotype **	**Source**
Strains		
*E. coli *TG1	*supE thi-1 *Δ(*lac-proAB*) Δ*(mcrB-hsdSM)5 *(r_K _^- ^m_K _^-^) [F' *traD36 proAB lacI*^q^ZΔM15]	[51]
*E. coli *MC4100	F- *araD139 *Δ*lacU169 rpsL thi fla*	[52]
*E. coli *HD705	MC4100 Δ*hycE*; defective in large subunit of the hydrogenase 3 subunit	[14]
*E. coli *SE1174	*thi-1 leu-6 suc-10 bioA2? galT27 rpsL129 chlC3 *λ- *fhlA102*::Tn10; defective transcription of the *hyc *operon and *fdhF*	[53]
*E. coli *SE1497	*cysC43 srl-300*::Tn10 *thr-1 leu-6 thi-1 proA2 galK2 ara-14 xyl-5 mtl-1 lacY1 his-4 argE3 rpsL31 tsx-33 *Δ(*srl-fhlA*); large deletion in the 59 min region removes the *hyc*, *hyp *or *fhlA *genes	[54]
*E. coli *PMD23	MC4100 Δ*hypF *; defective in full maturation of hydrogenase	K. T. Shanmugam
*E. coli *WL400	*selD *(Cm^R^)	[55]
*E. coli *BW25113	*lacI*^q^*rrnB*_T14_*ΔlacZ*_WJ16_*hsdR514 ΔaraBAD*_AH33_*ΔrhaBAD*_LD78_	Yale CGSG Stock Center
*E. coli *BW25113 *ΔhyaA Δkan*	*E. coli *JW0954 [41] *Δkan*; defective in small subunit of hydrogenase 1	this study
*E. coli *BW25113 *ΔhyaB Δkan*	*E. coli *JW0955 [41]*Δkan*; defective in large subunit of hydrogenase 1	this study
*E. coli *BW25113 *ΔhybB Δkan*	*E. coli *JW5494 [41] *Δkan*; defective in probable cytochrome Ni/Fe component of hydrogenase 2	this study
*E. coli *BW25113 *ΔhybC Δkan*	*E. coli *JW2962 [41]*Δkan*; defective in probable large subunit of hydrogenase 2	this study
*E. coli *BW25113 *ΔhycE Δkan*	*E. coli *JW2691 [41] *Δkan*; defective in large subunit of hydrogenase 3	this study
*E. coli *BW25113 *ΔhycG Δkan*	*E. coli *JW2689 [41]*Δkan*; defective in subunit of hydrogenase 3 and formate hydrogenlyase complex	this study
*E. coli *MW1000	BW25113 *ΔhyaB ΔhybC Δkan*; defective in large subunit of hydrogenase 1 and 2	this study
*E. coli *MW1001	BW25113 *ΔhyaB ΔhybC ΔhycE Δkan*; defective in large subunit of hydrogenases 1, 2, and 3	this study
*E. aerogenes *HU-101	Hydrogen-producing bacteria (positive control strain; wild type)	NBRC
Plasmids		
pBS(Kan)Synhox	pBS(Kan) *plac*::*hoxEFUYH*; Km^R^, expresses hydrogenase genes derived from *Synechocystis *sp. PCC 6803	this study
pBS(Kan)HoxU	pBS(Kan) *plac*::*hoxU*; Km^R^, expresses HoxU derived from *Synechocystis *sp. PCC 6803	this study
pBS(Kan)HoxEFU	pBS(Kan) *plac*::*hoxEFU*; Km^R^, expresses HoxEFU derived from *Synechocystis *sp. PCC 6803	this study
pBS(Kan)HoxFU	pBS(Kan) *plac*::*hoxFU*; Km^R^, expresses HoxFU derived from *Synechocystis *sp. PCC 6803	this study
pBS(Kan)HoxFUY	pBS(Kan) *plac*::*hoxFUY*; Km^R^, expresses HoxFUY derived from *Synechocystis *sp. PCC 6803	this study
pBS(Kan)HoxUYH	pBS(Kan) *plac*::*hoxUYH*; Km^R^, expresses HoxUYH derived from *Synechocystis *sp. PCC 6803	this study
pCP20	Ap^R ^and Cm^R ^plasmid with temperature-sensitive replication and thermal induction of FLP synthesis	[43]

### Eliminating kanamycin resistance and P1 transduction

Plasmid pCP20 [43] was used as described previously [44] to eliminate the kanamycin resistance gene (*kan*^R^) from the isogenic BW25113 mutants (Keio strains) defective in hydrogenase 1, hydrogenase 2, and hydrogenase 3 so that pBS(Kan)Synhox could be added and so that a double and triple mutant could be constructed (Table 3). P1 transduction [45] and pCP20 were used to create *E. coli *MW1000 (*hyaB hybC Δkan*) from BW25113 *hybC Δkan *by transferring *hyaB kan*^R ^via P1 transduction and using pCP20 to eliminate the kanamycin resistance marker. Similarly, MW1001 (*hyaB hybC hycE Δkan*) was created from BW25113 *hyaB hybC Δkan *by transferring *hycE kan*^R ^and then eliminating the kanamycin resistance marker.

### Constructing pBS(Kan)Synhox

*Synechocystis *sp. PCC 6803 genomic DNA was obtained using an UltraClean Microbial DNA Isolation Kit (Mo Bio Laboratories, Solana Beach, CA). The 6500 bp chromosomal DNA fragment encoding *hoxEFUYH *was amplified using *Taq *and *Pfu *polymerases mixture (1:1) using primers SynHoxEcoR1 Front [5'-CCAATCATGAATTCGCTGTATTGCTCCTTTTTGAGG-3'] and SynHoxXbaI Rear [5'-GACATTGAGTTCTTCTAGATATGCCTCGGTG-3'] with 30 cycles and 55°C annealing. The PCR product was cloned into the multiple cloning site in pBS(Kan) [42] after double digestion with *Xba*I and *Eco*RI to create pBS(Kan)Synhox (Figure 1A). Plasmid DNA was isolated using a Midi or Mini Kit (Qiagen, Inc., Chatsworth, CA), and polymerase chain reaction (PCR) products were purified with a Wizard^® ^PCR Preps DNA Purification System (Promega Corporation, Madison, WI). The correct plasmid was verified by digesting the plasmid with the restriction enzymes *Eco*RI/*Xba*I, *Avr*II, *Mfe*I/*Sna*BI, and *Pfl*MI. In addition, the beginning of *hoxE *and the end of *hoxH *of the cloned loci were sequenced [23] to show the presence of the *hoxEFUYH *locus using primers 5'-GACCATGATTACGCCAAGCGCGC-3' and 5'-GGGCGAATTGGAGCTCC-3', respectively.

### Constructing pBS(Kan)HoxU, pBS(Kan)HoxEFU, pBS(Kan)HoxFU, pBS(Kan)HoxFUY and pBS(Kan)UYH

The 803 bp, 3804 bp, 3113 bp, 3705 bp, and 3811 bp DNA fragments for *hoxU, hoxEFU, hoxFU, hoxFUY*, and *hoxUYH *were amplified from pBS(Kan)Synhox using *Pfu *polymerase with primers for HoxU (HoxUKpnI Front 5'-ACAATTTAGGTACCTCATTAACAAAGGAGTTTTTGGCCAATGTC-3' and HoxUEcoRI Rear 5'-ATGATTAAGAATTCAGAAATGATGTTAAAAGTTC-3'), for HoxEFU (HoxEFU Front 5'-ACAGCTATGACCATGATTACGCC-3' and HoxEFUXbaI Rear 5'-TAGCCATGTCTAGAAGTTTAGAAATGATGTTAAAAG-3'), for HoxFU (HoxFUKpnI Front 5'-TCTAGTTGGGTACCTGATTAATTGTTAAGGAGGTTAAACCCCATGGAC-3' and HoxFUEcoRI Rear 5'-ATGATTAAGAATTCAGAAATGATGTTAAAAGTTC-3'), for HoxFUY (HoxFUYKpnI Front 5'-TCTAGTTGGGTACCTGATTAATTGTTAAGGAGGTTAAACCCCATGGAC-3' and HoxFUYXbaI Rear [5'-ATCTCCTGTCTAGATATTTTGCAAACTGTTTAG-3'), and for HoxUYH (HoxUYHKpnI Front 5'-ACAATTTAGGTACCTCATTAACAAAGGAGTTTTTGGCCAATGTC-3'] and HoxUYH Rear [5'-AAGGCGAT TAAGTTGGGTAACGC-3') with 30 cycles and 54°C annealing. The PCR products were cloned into the multiple cloning site in pBS(Kan) [42] after double digestion with *Kpn*I and *Eco*RI to create pBS(Kan)HoxU (Figure 1B), with *Eco*RI and *Xba*I to create pBS(Kan)HoxEFU (Figure 1C), with *Kpn*I and *Eco*RI to create pBS(Kan)HoxFU (Figure 1D), with *Kpn*I and *Xba*I to create pBS(Kan)HoxFUY (Figure 1E), and with *Kpn*I and *Xba*I to create pBS(Kan)HoxUYH (Figure 1F). The correct plasmids were verified by digesting the plasmid with the restriction enzymes *Kpn*I/*Bam*HI, *Ava*I, *Ban*I, *Dra*I, *Pvu*I and *Pvu*II for pBS(Kan)HoxU, *Kpn*I/*Xba*I, *Bsp*HI, *Nco*I, *Xho*I, *Pvu*II and *Apa*I for pBS(Kan)HoxEFU, *Kpn*I/*Sac*I, *Bsp*HI, *Pst*I, *Pvu*II, *Nco*I and *Apa*I for pBS(Kan)HoxFU, *Kpn*I/*Sac*I, *Bsp*HI, *Pvu*II, *Nco*I and *Apa*I for pBS(Kan)HoxFUY, and *Kpn*I/*Sac*I, *Bsp*HI, *Ava*I, *Bcl*I and *Pvu*II for pBS(Kan)HoxUYH.

### Hydrogen assay and SDS-PAGE

Overnight, aerobic cultures (25 mL) were used to inoculate 75 mL of the complex medium (with 5 mM IPTG for the *E. coli *strains) in 250 mL shake flasks, and these cultures were sparged for 5 min with nitrogen, sealed, and incubated anaerobically at 37°C for 6 h to induce expression of the cyanobacterial hydrogenase system. After 6 h the cultures were poured anaerobically into a 250 mL centrifuge tubes in a glove box, and centrifuged (7350 × *g*) for 10 min at 4°C. The supernatant was decanted in the glove box, and 25 mL of complex medium (including 5 mM IPTG for *E. coli *cells) was added. Sealed crimp-top vials (27 mL) were sparged for 5 min with nitrogen, and 20 mL of the cell suspension was added to the bottles which were incubated at 37°C with shaking for 1.5 to 24 h. The amount of hydrogen generated in the head space of the recombinant system was measured using a 50 μL of aliquot by gas chromatography (GC) using a 6890 N gas chromatograph (Agilent Technologies, Glastonbury, CT) equipped with a 80–100 mesh Porapak Q column (Suppelco, Bellefonte, PA) and a thermal conductivity detector. The injector and detector were maintained at 100°C and 200°C respectively. The nitrogen carrier gas flow rate was maintained at 20 mL/min. The column temperature was 70°C. Under these conditions, the retention time for hydrogen was 0.38 min and the sensitivity is about 0.1 μmol. Retention times were determined by comparisons to neat standards as well as by co-elution with standards. Hydrogen productivity was calculated as μmol H_2_/mg total protein. Expression of recombinant proteins from samples actively expressing hydrogen was analyzed with standard Laemmli discontinuous SDS-PAGE (12%) [40].

### Hydrogen uptake assays

Hydrogen uptake activities were measured as the increase in absorbance as oxidized methylviologen (MV) (ε_604 _= 13.9 mM^-1 ^cm^-1^[21]) is reduced [MV^+2 ^+ 1/2H_2_→ MV^+1 ^+ H^+^] as reported previously [46] except whole cells were used rather than lysed cells. Cells were prepared as for the hydrogen assay except 1 mM IPTG was added to induce expression of the cyanobacterial hydrogenase system for 6 h, then the cell pellets from 1.5 mL were resuspended with 1 mL Tris buffer (50 mM, pH 8.0) in the anaerobic glove box. Oxidized MV (MV^+2^, colorless) solution (1 mL, 0.8 mM in 50 mM Tris buffer, pH 8) was sparged first with nitrogen gas for 10 min to remove oxygen to prevent residual oxygen from oxidizing any reduced MV (MV^+1^, purple) that is formed by the hydrogenases, was poured into cuvettes that were sealed with rubber stoppers, and was sparged with pure hydrogen gas for 10 min. Whole cell suspensions (0.5 mL) were mixed into the cuvettes and the change in absorbance during 5 min was monitored using a spectrophotometer (Varian, Walnut Creek, CA). Two independent cultures were used.

To corroborate the MV uptake assay, a filter paper assay for reversible hydrogenase activity was used based on a method described previously [47]. Filter paper (Whatman 541; Whatman plc, 27 Great West Road, UK) was immersed in MV solution (18 mM in 6 mM Tris buffer, pH 7.5), was air-dried with a hair dryer, and was firmly pressed on agar plates containing equal-size of colonies. The filter paper was then incubated at room temperature in a moist atmosphere of pure hydrogen in a Gas-Pak anaerobic chamber. The cells and adjacent white filter turned from white to blue-purple in approximately 10 min.

For the GC-based hydrogen uptake assay, cells were prepared as for the hydrogen assay except 1 mM IPTG was added to induce expression of the HoxEFUYH enzymes and after 6 hours, the cell pellets from 100 mL were resuspended with 25 mL phosphate buffer (100 mM, pH 7.5) including 1 mM IPTG in the anaerobic glove box. The cell suspension (20 mL) was added to sealed crimp-top vials (27 mL). The bottles were sparged with hydrogen for 10 min, were incubated at 37°C with shaking for 3 to 6 h, and the amount of hydrogen in the head space was measured as described above.

### Microarray analysis

To isolate RNA from hydrogen producing cells, TG1/pBS(Kan)Synhox and TG1/pBS(Kan) were cultured in complex medium with fructose as for the hydrogenase assay. RNA was isolated as described previously [48] with the RNeasy kit (Qiagen, Inc.). To inhibit RNase and ensure high-quality RNA, β-mercaptoethanol, which acts as a reducing agent to irreversibly denature RNase, and guanidinium isothiocyanate contained in the RLT buffer (RNA Lysis Tissue, RNeasy mini kit: Qiagen, Inc.), which is a strong but temporary RNase-denaturing agent, were utilized. The *E. coli *GeneChip antisense genome array was used (part no. 900381, Affymetrix, Inc., Central Expressway, Santa Clara, CA), and contains probe sets for all 4,290 open reading frames, rRNA, tRNA, and 1,350 intergenic regions. Analysis of the microarray data was as previously described [32], and the data have been deposited in the NCBI Gene Expression Omnibus [49] and are accessible through accession numbers GSM129630 and GSM129631.

### Ninety-six-well biofilm assay

Biofilm formation was quantified in 96-well polystyrene plates as reported previously [50]. Biofilm formation of *E. coli *TG1 with pBS(Kan)Synhox and pBS(Kan) was measured in LB supplemented with 0.2% glucose under anaerobic conditions using a Gas-Pak system. Thirty replicate wells were averaged to obtain each data point. Three independent cultures were used.

## Authors' contributions

TM performed all the experiments except for constructing pBS(Kan)Synhox which was made by GV. WTS suggested some experiments with hydrogenase mutants, and TKW contributed to experimental design, authored some of the manuscript, and managed the project. All authors read and approved the final manuscript.

## Supplementary Material

Additional file 1 Microarray data for induced and repressed genes upon expressing the cyanobacterial locus.  The data indicate differential gene expression in *Escherichia coli* TG1 cells upon expressing the cyanobacterial hydrogenase locus *hoxEFUYH* derived from *Synechocystis* sp. PCC6803. Click here for file
