# Supplementary material for: Inhibition of hydrogen uptake in Escherichia coli by expressing the hydrogenase from the cyanobacterium Synechocystis sp. PCC 6803
Source: BMC Biotechnol. 2007 May 23;7:25. doi: 10.1186/1472-6750-7-25 (PMC1904212; doi:10.1186/1472-6750-7-25)
Supplement: Additional file 1 — Microarray data for induced and repressed genes upon expressing the cyanobacterial locus. The data indicate differential gene expression in Escherichia coli TG1 cells upon expressing the cyanobacterial hydrogenase locus hoxEFUYH derived from Synechocystis sp. PCC6803. [file 1472-6750-7-25-S1.pdf]

4/3/2006, Toshinari Maeda, Wood lab, TAMU

Microarray analysis of TG1/pBS(Kan)Synhox vs. TG1/pBS(Kan)

Objectives

To investigate whether:

1. there is a difference in metabolic activity upon expressing Synhox by using *E. coli* TG1/pBS(Kan)Synhox and TG1/pBS(Kan) when both strains produce hydrogen [*E. coli* TG1/pBS(Kan)Synhox produces 3-fold more hydrogen than TG1/pBS(Kan)]

2. cloning of the cyanobacterium *hoxEFUYH* merely increases hydrogen production by up-regulating the native *E. coli* hydrogenase system

Conclusions

1. Gene expression for the *hya, hyb, hyc,* and *hyp* operons was not altered significantly between TG1/pBS(Kan)Synhox and TG1/pBS(Kan); namely, functional HoxEFUYH is necessary for enhanced hydrogen production in *E. coli.*

2. Using a cut-off ratio of 5.3 (roughly the standard deviation for all the genes), 33 genes were induced and 17 were repressed that are related to other aspects of cell metabolism.

Strains

1. *E. coli* TG1/pBS(Kan)Synhox
2. *E. coli* TG1/pBS(Kan)

Procedures

1. Single colonies from one day-old streaked cultures were used to inoculate complex medium (without glucose but with fructose) and allowed to incubate aerobically at 37 ^{o}C overnight for the two strains.
2. The aerobic overnight cultures, fresh medium (including 5 mM IPTG), and sealed crimp-top vials (27 mL) were sparged for 5 min with nitrogen, and then 19 mL of fresh medium and the overnight culture (1 mL, sparged) were mixed into sealed crimp-top vials in a glove box.
3. These vials were anaerobically incubated at 37 ^{o}C for 6 hour. After 6 hour, these cultures were used for isolation of total DNA. Cell cultures (1.8 mL) were collected into chilled bead beater vials using a syringe.

Table 1. Induced genes for TG1/pBS(Kan)Synhox vs. TG1/pBS(Kan).

Gene name	b-#	Signal	Detection	Fold change	Base line signal	Base line detection	Gene function
**Amino acid catabolism genes (tryptophan)**							
tnaL	b3707	13938.7	P	24.3	549.7	P	tryptophanase leader peptide
tnaA	b3708	3354.3	P	13.9	244.7	P	subunit of L-cysteine desulfhydrase / tryptophanase
**Amino acid catabolism genes (threonine catabolism)**							
tdcB	b3117	1611.8	P	9.8	195.1	P	subunit of threonine dehydratase (catabolic)
tdcC	b3116	1591.3	P	8.6	182.4	P	TdcC threonine STP transporter
tdcD	b3115	1278.8	P	8.0	202.6	P	propionate kinase / acetate kinase C
tdcE	b3114	2218.4	P	6.5	351.3	P	2-ketobutyrate formate-lyase / pyruvate formate-lyase 4
**Prophage genes and phage related function genes**							
ydfX	b1568	193.2	P	34.3	6.4	A	hypothetical protein
pspA	b1304	4034.6	P	9.2	589	P	regulatory protein for the phage shock protein operon
pspB	b1305	6335	P	8.0	753.8	P	stimulates PspC-mediated transcriptional activation of the *psp* operon
pspC	b1306	1971.6	P	5.7	380.6	P	PspC transcriptional regulator
nmpC	b0553	1154.1	P	6.1	208.5	P	outer membrane porin protein
**Energy production/transport (electron donor) genes**							
sdhC	b0721	1438.1	P	10.6	154	P	succinate dehydrogenase membrane protein
sdhD	b0722	2005.7	P	9.2	186.9	P	succinate dehydrogenase membrane protein
**Energy metabolism (TCA cycle) genes**							
gltA	b0720	2226.6	P	8.0	335	P	subunit of citrate synthase
mdh	b3236	2383.6	P	5.7	424.7	P	subunit of malate dehydrogenase
sucC	b0728	2869.6	P	5.3	454.7	P	succinyl-CoA synthetase, β subunit
**Phosphotransferase System genes**							
treB	b4240	2321.8	P	9.2	380.3	P	subunit of EIITre

treC	b4239	1181.6	P	6.1	174.2	M	trehalose-6-phosphate hydrolase
gatA	b2094	14585.4	P	6.1	2506.2	P	subunit of EIIGat
gatB	b2093	8325.5	P	7.5	1325.5	P	subunit of EIIGat
**Transporter genes**							
glpT	b2240	4895.1	P	6.5	767.6	P	glycerol-3-P MFS transporter
yhaM	b3108	2518.2	P	5.3	727.3	P	hypothetical ORF
yhaN	b3109	5431.5	P	8.6	605.3	P	hypothetical ORF
yhaO	b3110	3790.1	P	9.8	411	P	YhaO STP transporter
yhaR	b3113	1558.9	P	5.7	277.2	P	hypothetical ORF
**Adaptations (temperature extremes) genes**							
ibpB	b3686	7293.4	P	6.5	1220.5	P	small heat shock protein IbpB
**Sulfur metabolism gene**							
ydeN	b1498	1434.2	P	6.5	214.4	P	putative sulfatase
**Outer membrane protein gene**							
yciD	b1256	1787.5	P	8.0	256.3	P	subunit of Colicin S4 Transport System
**tRNA genes**							
valT	b0744	17792	P	6.1	2784.7	P	tRNA for Val (one of seven valine tRNAs)
valZ	b0746	14873.2	P	5.7	2452.4	P	tRNA for Val (one of seven valine tRNAs)
valX	b2402	14196.8	P	5.3	2416.2	P	tRNA for Val (one of seven valine tRNAs)
glyW	b1911	4921	P	5.3	803.5	P	tRNA for Gly (one of six glycine tRNAs)
glyV	b4163	6106.8	P	5.3	1243.4	P	tRNA for Gly (one of six glycine tRNAs)
**lactose related genes**							
lacY	b0343	3409.5	P	5.7	612.3	P	LacY lactose MFS transporter
lacA	b0342	2377.4	P	5.7	391.7	P	subunit of galactoside O-acetyltransferase

Table 2. Repressed genes for TG1/pBS(Kan)Synhox vs. TG1/pBS(Kan)

Gene name	b-#	Signal	Detection	Fold change	Base line signal	Base line detection	Gene function
**Two component regulatory system gene**							
torT	b0994	33.5	A	-3.0	134.4	P	TorT-unknown inducer
**Electrochemical potential driven transporter genes**							
yicE	b3654	36.4	A	-5.3	248.3	P	YicE NCS2 transporter
yihP	b3877	31.1	A	-8.0	236.7	P	YihP GPH transporter
melB	b4120	6.5	A	-36.8	329.6	P	MelB GPH transporter
**Prophage genes and phage related function genes**							
yfjT	b2637	9.6	A	-7.5	181.6	P	CP4-57 prophage; predicted protein
intB	b4271	25.5	A	-9.2	212.8	P	KpLE2 phage-like element; predicted integrase
**Transcription related regulation gene**							
ydeO	b1499	9	A	-10.6	118.8	P	YdeO transcriptional activator
**ATP proton motive force interconversion gene**							
atpC	b3731	23.7	A	-12.1	724.9	P	ATP synthase, F1 complex, ε subunit
**ABC transporter gene**							
yjfF	b4231	6.5	A	-34.3	190.7	P	subunit of YtfQ/YtfR/YtfS/YtfT/YjfF ABC transporter
**TCA cycle gene**							
fumB	b4122	19	A	-14.9	397.6	P	subunit of fumarase B
**DNA degradation gene**							
mcrB	b4346	8.1	A	-32.0	229.5	P	MrcB subunit of 5-methylcytosine restriction system
**Unassigned reversible reaction gene**							
sgaE	b4198	21.1	A	-18.4	295.5	P	L-ribulose 5-phosphate 4-epimerase
**Unknown function genes**							

yghG	b2971	18.6	A	-5.3	92.7	P	unknown function
yjhS	b4309	12.3	A	-6.5	119.8	P	conserved protein
yjeN	b4157	13.8	A	-8.0	163.6	P	conserved protein
yjjJ	b4385	15.6	A	-9.2	196.1	P	conserved protein
yigE	b3814	15.9	A	-11.3	228.7	P	conserved protein

Table 3. Gene expression for *E. coli* native hydrogenase 3 and the related proteins (formate hydrogenase H and maturation protein) upon cloning *hoxEFUYH.*

Gene name	pBS(Kan) signal	Synhox signal	Fold change	Gene function
hycA	2538.2	1820.7	-1.3	Transcriptional repression of hyc and hyp operon
hycB	2589.3	1658.9	-1.6	Probable small subunit of hydrogenase 3
hycC	1099.5	1218.6	1.2	Membrane-spanning protein of hydrogenase 3
hycD	827.8	1193.9	1.3	Membrane-spanning protein of hydrogenase 3
hycE	827.8	1114.5	1.3	Large subunit of hydrogenase 3
hycF	1844.1	2699.4	1.4	Iron-sulfur protein of hydrogenase 3
hycG	895.7	1215.8	1.4	Hydrogenase activity
hycH	894.7	1583	1.6	Processing of large subunit (HycE) of hydrogenase 3
hycI	1128.4	1344	1.2	Protease involved in processing C-terminal end of HycE
hypA	2381.9	1545.1	-1.4	Pleiotrophic effects on 3 hydrogenase isozyme
hypB	3698	2130.2	-1.7	Guanosine-nucleotide binding protein, functions as nickel-donor for HycE
hypC	2569.6	2331.4	-1.3	Pleiotrophic effects on 3 hydrogenase isozyme
hypD	2763.7	2032.8	-1.4	Pleiotrophic effects on 3 hydrogenase isozyme
hypE	1297	1176.8	1	Plays structural role in maturation of all 3 hydrogenase
hypF	389.6	521.7	1	Transcriptional regulatory protein
fhlA	493.9	715.1	1.3	Transcriptional activator for fdhF, hyc and hyp operon
fdhF	2346.7	1193.9	-1.7	Selenopolypeptide subunit of formate dehydrogenase H

Table 4. Gene expression for *E. coli* native hydrogenase 1 and hydrogenase 2 upon cloning *hoxEFUYH*.

Gene name	pBS(Kan) signal	Synhox signal	Fold change	Gene function
hyaA	5877.9	5768	1.1	Small subunit of hydrogenase 1
hyaB	3413.7	3011.6	-1.1	Large subunit of hydrogenase 1
hyaC	3199.5	2781.5	-1.1	Probable Ni/Fe-hydrogenase 1 b-type cytochrome subunit
hyaD	2942	2740.4	-1.1	Processing of HyaA and HyaB
hyaE	1884.8	1701.3	-1.1	Processing of HyaA and HyaB
hyaF	908.7	1071.7	1.3	Nickel incorporation into hydrogenase 1 protein
hybA	1796	2617.8	1.4	hydrogenase 2 4Fe-4S ferredoxin-type component
hybB	557.8	870.8	1.4	Probable cytochrome Ni/Fe component of hydrogenase 2
hybC	365.6	566.6	1.5	large subunit of hydrogenase 2
hybD	522.5	489.5	-1.1	Probable processing element for hydrogenase 2
hybE	513.2	617.8	1.3	Member of hyb operon
hybF	270	380.6	1.3	may modulate levels of hydrogenase 2
hybG	708.6	663.7	1	Hydrogenase 2 operon protein: may effect maturation of large subunit of hydrogenase 2
